# Supplementary material for: Crafting a Personalized Prognostic Model for Malignant Prostate Cancer Patients Using Risk Gene Signatures Discovered through TCGA-PRAD Mining, Machine Learning, and Single-Cell RNA-Sequencing
Source: Diagnostics (Basel). 2023 Jun 7;13(12):1997. doi: 10.3390/diagnostics13121997 (PMC10297172; doi:10.3390/diagnostics13121997)
Supplement: Supplementary file 1 [file diagnostics-13-01997-s001.zip › Figure S1. Expression analysis of 5 risk factors in cellular subgroups of prostate cancer.pdf]

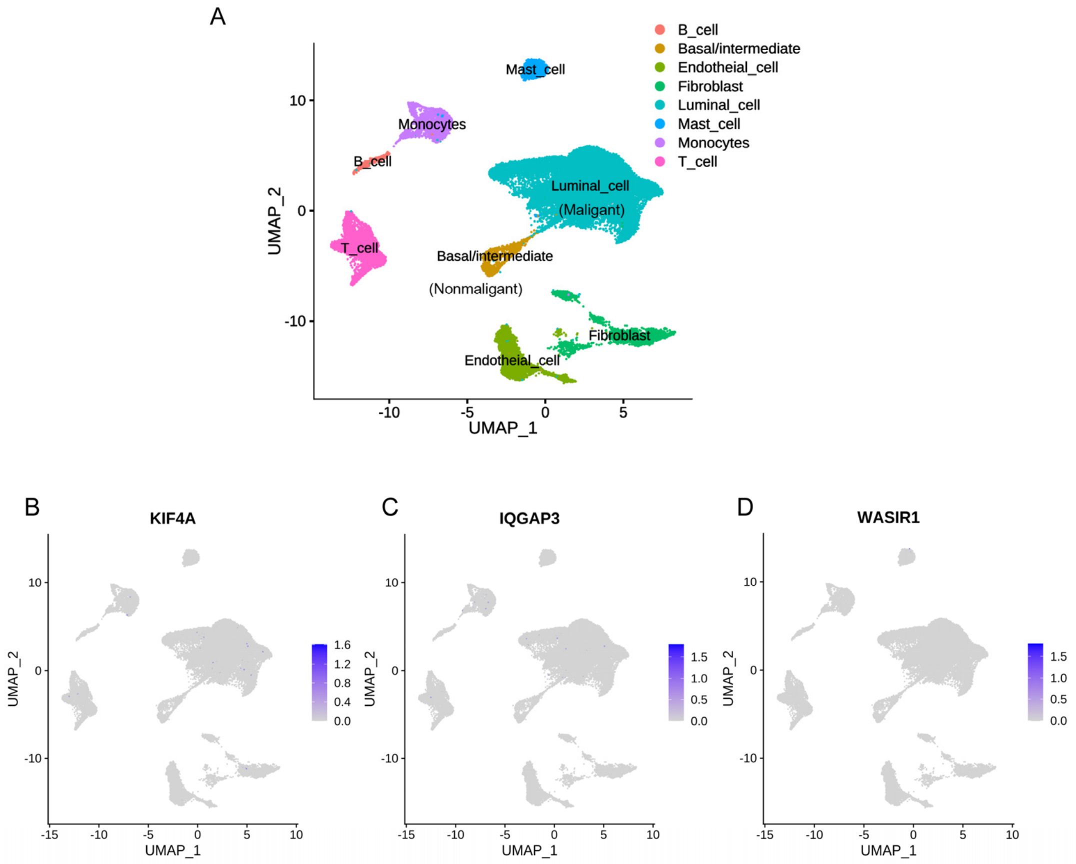

Figure S1. Expression analysis of 5 risk factors in cellular subgroups of prostate cancer. (A) The annotation of cell subpopulations. Analysis of the expression of KIF4A (B), IQGAP3 (C), and WASIR1 (D) in cellular subgroups using the GSE 141445 dataset.
