# Supplementary material for: Crafting a Personalized Prognostic Model for Malignant Prostate Cancer Patients Using Risk Gene Signatures Discovered through TCGA-PRAD Mining, Machine Learning, and Single-Cell RNA-Sequencing
Source: Diagnostics (Basel). 2023 Jun 7;13(12):1997. doi: 10.3390/diagnostics13121997 (PMC10297172; doi:10.3390/diagnostics13121997)
Supplement: Supplementary file 1 [file diagnostics-13-01997-s001.zip › Table S1. Clinical Information of PCa Patients in TCGA-PRAD.pdf]

Table S1. Clinical Information of PCa Patients in TCGA-PRAD

Characteristic	levels	Overall
n		499
T stage, n (%)	T2	189 (38.4%)
	T3	292 (59.3%)
	T4	11 (2.2%)
N stage, n (%)	N0	347 (81.5%)
	N1	79 (18.5%)
M stage, n (%)	M0	455 (99.3%)
	M1	3 (0.7%)
Race, n (%)	Asian	12 (2.5%)
	Black or African American	57 (11.8%)
	White	415 (85.7%)
Primary therapy outcome, n (%)	PD	28 (6.4%)
	SD	29 (6.6%)
	PR	40 (9.1%)
	CR	341 (77.9%)
Age, n (%)	<=60	224 (44.9%)
	>60	275 (55.1%)
Residual tumor, n (%)	R0	315 (67.3%)
	R1	148 (31.6%)
	R2	5 (1.1%)
Zone of origin, n (%)	Central Zone	4 (1.5%)
	Overlapping / Multiple Zones	126 (45.8%)
	Peripheral Zone	137 (49.8%)
	Transition Zone	8 (2.9%)
PSA(ng/ml), n (%)	<4	415 (93.9%)
	>=4	27 (6.1%)
Gleason score, n (%)	6	46 (9.2%)
	7	247 (49.5%)
	8	64 (12.8%)
	9	138 (27.7%)
	10	4 (0.8%)
Age, median (IQR)		61 (56, 66)
PSA(ng/ml), median (IQR)		0.1 (0.03, 0.11)
