# Supplementary material for: Crafting a Personalized Prognostic Model for Malignant Prostate Cancer Patients Using Risk Gene Signatures Discovered through TCGA-PRAD Mining, Machine Learning, and Single-Cell RNA-Sequencing
Source: Diagnostics (Basel). 2023 Jun 7;13(12):1997. doi: 10.3390/diagnostics13121997 (PMC10297172; doi:10.3390/diagnostics13121997)
Supplement: Supplementary file 1 [file diagnostics-13-01997-s001.zip › Table S2. Primer Sequences used for Real-time PCR.pdf]

Table S2. Primer Sequences used for Real-time PCR

Primer	Sequnce (5'→ 3')
WASIR1-F	ATTCTCCACGGCTGCCATTA
WASIR1-R	AAACTCCGTCCTCACAGTCC
KRTAP5-1-F	TGCCCGTGTGTTGCTGTTCCTG
KRTAP5-1-R	ATGACCCACAGCCTGAGGAACA
TLX1-F	GGTCAAAACCTGGTTCCAGAACC
TLX1-R	TGTGCCAGGCTCTTCTGGAAGG
IQGAP3-F	GTTCGGCAGAAGTTTGCTGAGC
IQGAP3-R	CACTCCAGGTAAATCTTCCGCTG
U6-F	CTCGCTTCGGCAGCACATATACT
U6-R	CGCTTCACGAATTTGCGTGT
RPL19-F	AAGCCTGTGACGGTCCATTC
RPL19-R	TGGCTGTACCCTTCCGCTTA
