# Supplementary material for: Crafting a Personalized Prognostic Model for Malignant Prostate Cancer Patients Using Risk Gene Signatures Discovered through TCGA-PRAD Mining, Machine Learning, and Single-Cell RNA-Sequencing
Source: Diagnostics (Basel). 2023 Jun 7;13(12):1997. doi: 10.3390/diagnostics13121997 (PMC10297172; doi:10.3390/diagnostics13121997)
Supplement: Supplementary file 1 [file diagnostics-13-01997-s001.zip › Table S3. Research Progress on Risk Stratification and Treatment Outcome Assessment Models for Prostate Cancer.pdf]

Table S3. Research Progress on Risk Stratification and Treatment Outcome Assessment Models for Prostate Cancer

Model Name	Time and Institution	Contents	Detection Efficacy	Purpose	Existing Issues
Decipher Gene Classifier (GC) [1]	Genome Dx Biosciences, Vancouver, Canada, 2015.	Evaluation of RNA expression levels of 22 biomarkers in prostate cancer tissue samples, risk assessment scored from 0-1.	AUC: 0.64-0.72; C-index: 0.71 for predicting risk of distant metastasis 5 years after biopsy [2-4].	Recommended by NCCN guidelines for risk stratification of prostate cancer following radical prostatectomy, predicting 10-year risk of distant metastasis, and guiding postoperative radiation dosage based on latest research as of 2022.	Multiple biomarkers are involved and High testing thro ughput.
Prolaris (CCP Score) [5-7]	Research on breast cancer started in 2002; Prostate cancer-related research conducted by Myriad Genetics, Salt Lake City in 2014.	Tests 31 cell cycle-related genes and 15 housekeeping genes in prostate tissue to evaluate related risks using a scoring system from 1.3 to 4.7.	For every one point increase in CCP score after surgery, there is a 1.89-fold increase in the risk of biochemical recurrence [8,9].	Recommended by NCCN guidelines for predicting distant metastasis rate and mortality in patients post-prostatectomy. Also being evaluated for assessing the efficacy of radiotherapy.	The efficacy of this model has not yet been validated by prospective randomized controlled trials.
OncotypeDX® Genomic Prostate Score (GPS) [10]	Developed by Genomic Health, Redwood City, USA in 2013 [11].	Tests the expression of 17 genes in prostate cancer tissue samples that are involved in 4 pathways: androgen receptor, cell proliferation, cellular and extracellular matrix.	For every 20-point increase in GPS score, there is a 1.9-fold increase in the risk of distant metastasis.	Recommended by NCCN guidelines to help assess patient prognosis and risk stratification.	-

Contimued Table S3. Research Progress on Risk Stratification and Treatment Outcome Assessment Models for Prostate Cancer

Model Name	Time and Institution	Contents	Detection Efficacy	Purposes	Existing Issues
ProMark [12]	2014, Metamark Genetics, USA.	Used immunofluorescence to detect expression of 8 protein molecules in prostate cancer tissues, scored from 0-1.	Predictive value of 95%	Recommended in the NCCN guidelines for prognostic risk stratification	No relevant prospective randomized controlled trials have yet validated the predictive power of this model.
ADT-RS [13]	2018, Mayo Clinic, USA.	Selected 49 relevant genes from the Decipher GRID database to predict response to ADT treatment.	Single-center study	Patients with higher ADT-RS scores can benefit from ADT treatment; these patients experience a decrease in distant metastasis rates after receiving ADT.	Lacks multicenter, prospective data to confirm.
PAM50 [14,15]	Started in breast cancer research in 2009, and transitioned to prostate cancer by the University of Michigan research team in 2017	Using the expression of 50 PAM50 genes and 5 control genes in surgical specimens, prostate cancer is classified into Lum A, Lum B, and Basal subtypes for molecular typing.	Single-center study	Luminal B subtype can benefit from postoperative ADT, while the benefits for other subtypes are not significant.	Recent studies have shown that molecular typing can be performed in patients with mCRPC.
RSI [16]	Conducted by the National Cancer Institute (NCI) in the US in 2012	11 relevant genes were screened from the molecular expression profiles of over 60 irradiated cells.	-	It can predict the sensitivity of prostate cancer patients to radiotherapy.	It cannot predict the outcome of radiotherapy for prostate cancer patients; the lack of validation by clinical trials.

References

1. Erho, N.; Crisan, A.; Vergara, I.A.; Mitra, A.P.; Ghadessi, M.; Buerki, C.; Bergstralh, E.J.; Kollmeyer, T.; Fink, S.; Haddad, Z.; et al. Discovery and validation of a prostate cancer genomic classifier that predicts early metastasis following radical prostatectomy. *PLoS One* **2013**, *8*, e66855, doi:10.1371/journal.pone.0066855.
2. Feng, F.Y.; Huang, H.C.; Spratt, D.E.; Zhao, S.G.; Sandler, H.M.; Simko, J.P.; Davicioni, E.; Nguyen, P.L.; Pollack, A.; Efstathiou, J.A.; et al. Validation of a 22-Gene Genomic Classifier in Patients With Recurrent Prostate Cancer: An Ancillary Study of the NRG/RTOG 9601 Randomized Clinical Trial. *JAMA Oncol* **2021**, *7*, 544-552, doi:10.1001/jamaoncol.2020.7671.
3. Karnes, R.J.; Choeurng, V.; Ross, A.E.; Schaeffer, E.M.; Klein, E.A.; Freedland, S.J.; Erho, N.; Yousefi, K.; Takhar, M.; Davicioni, E.; et al. Validation of a Genomic Risk Classifier to Predict Prostate Cancer-specific Mortality in Men with Adverse Pathologic Features. *Eur Urol* **2018**, *73*, 168-175, doi:10.1016/j.eururo.2017.03.036.
4. Nguyen, P.L.; Haddad, Z.; Ross, A.E.; Martin, N.E.; Deheshi, S.; Lam, L.L.C.; Chelliserry, J.; Tosoian, J.J.; Lotan, T.L.; Spratt, D.E.; et al. Ability of a Genomic Classifier to Predict Metastasis and Prostate Cancer-specific Mortality after Radiation or Surgery based on Needle Biopsy Specimens. *Eur Urol* **2017**, *72*, 845-852, doi:10.1016/j.eururo.2017.05.009.
5. Arsov, C.; Jankowiak, F.; Hiester, A.; Rabenalt, R.; Quentin, M.; Schimmöller, L.; Blondin, D.; Antoch, G.; Albers, P. Prognostic value of a cell-cycle progression score in men with prostate cancer managed with active surveillance after MRI-guided prostate biopsy--a pilot study. *Anticancer Res* **2014**, *34*, 2459-2466.
6. Mosley, J.D.; Keri, R.A. Cell cycle correlated genes dictate the prognostic power of breast cancer gene lists. *BMC Med Genomics* **2008**, *1*, 11, doi:10.1186/1755-8794-1-11.
7. Whitfield, M.L.; Sherlock, G.; Saldanha, A.J.; Murray, J.I.; Ball, C.A.; Alexander, K.E.; Matese, J.C.; Perou, C.M.; Hurt, M.M.; Brown, P.O.; et al. Identification of genes periodically expressed in the human cell cycle and their expression in tumors. *Mol Biol Cell* **2002**, *13*, 1977-2000, doi:10.1091/mbc.02-02-0030.
8. Cuzick, J.; Swanson, G.P.; Fisher, G.; Brothman, A.R.; Berney, D.M.; Reid, J.E.; Mesher, D.; Speights, V.O.; Stankiewicz, E.; Foster, C.S.; et al. Prognostic value of an RNA expression signature derived from cell cycle proliferation genes in patients with prostate cancer: a retrospective study. *Lancet Oncol* **2011**, *12*, 245-255, doi:10.1016/s1470-2045(10)70295-3.
9. Freedland, S.J.; Gerber, L.; Reid, J.; Welbourn, W.; Tikishvili, E.; Park, J.; Younus, A.; Gutin, A.; Sangale, Z.; Lanchbury, J.S.; et al. Prognostic utility of cell cycle progression score in men with prostate cancer after primary external beam radiation therapy. *Int J Radiat Oncol Biol Phys* **2013**, *86*, 848-853, doi:10.1016/j.ijrobp.2013.04.043.
10. Knezevic, D.; Goddard, A.D.; Natraj, N.; Cherbavaz, D.B.; Clark-Langone, K.M.; Snable, J.; Watson, D.; Falzarano, S.M.; Magi-Galluzzi, C.; Klein, E.A.; et al. Analytical validation of the Oncotype DX prostate cancer assay - a clinical RT-PCR assay optimized for prostate needle biopsies. *BMC Genomics* **2013**, *14*, 690, doi:10.1186/1471-2164-14-690.
11. Klein, E.A.; Cooperberg, M.R.; Magi-Galluzzi, C.; Simko, J.P.; Falzarano, S.M.; Maddala, T.; Chan, J.M.; Li, J.; Cowan, J.E.; Tsiatis, A.C.; et al. A 17-gene assay to predict prostate cancer aggressiveness in the context of Gleason grade heterogeneity, tumor multifocality, and biopsy undersampling. *Eur Urol* **2014**, *66*, 550-560, doi:10.1016/j.eururo.2014.05.004.
12. Blume-Jensen, P.; Berman, D.M.; Rimm, D.L.; Shipitsin, M.; Putzi, M.; Nifong, T.P.; Small, C.; Choudhury, S.; Capela, T.; Coupal, L.; et al. Development and clinical validation of an in situ biopsy-based multimarker assay for risk stratification in prostate cancer. *Clin Cancer Res* **2015**, *21*, 2591-2600, doi:10.1158/1078-0432.Ccr-14-2603.
13. Karnes, R.J.; Sharma, V.; Choeurng, V.; Ashab, H.A.; Erho, N.; Alshalalfa, M.; Trock, B.; Ross, A.; Yousefi, K.; Tsai, H.; et al. Development and Validation of a Prostate Cancer Genomic Signature that Predicts Early ADT Treatment Response Following Radical Prostatectomy. *Clin Cancer Res* **2018**, *24*, 3908-3916, doi:10.1158/1078-0432.Ccr-17-2745.
14. Coleman, I.M.; DeSarkar, N.; Morrissey, C.; Xin, L.; Roudier, M.P.; Sayar, E.; Li, D.; Corey, E.; Haffner, M.C.; Nelson, P.S. Therapeutic Implications for Intrinsic Phenotype Classification of Metastatic Castration-Resistant Prostate Cancer. *Clin Cancer Res* **2022**, *28*, 3127-3140, doi:10.1158/1078-0432.Ccr-21-4289.
15. Zhao, S.G.; Chang, S.L.; Erho, N.; Yu, M.; Lehrer, J.; Alshalalfa, M.; Speers, C.; Cooperberg, M.R.; Kim, W.; Ryan, C.J.; et al. Associations of Luminal and Basal Subtyping of Prostate Cancer With Prognosis and Response to Androgen Deprivation Therapy. *JAMA Oncol* **2017**, *3*, 1663-1672, doi:10.1001/jamaoncol.2017.0751.
16. Torres-Roca, J.F. A molecular assay of tumor radiosensitivity: a roadmap towards biology-based personalized radiation therapy. *Per Med* **2012**, *9*, 547-557, doi:10.2217/pme.12.55.
